# Evaluation of Varian’s SmartAdapt for clinical use in radiation therapy for patients with thoracic lesions

**DOI:** 10.1002/acm2.13194

**Published:** 2021-02-11

**Authors:** Jason Vickress, Maria Alejandra Rangel Baltazar, Hossein Afsharpour

**Affiliations:** ^1^ Trillium Health Partners/the Credit Valley Hospital Mississauga ON Canada; ^2^ Department of Radiation Oncology University of Toronto Toronto ON Canada

**Keywords:** Deformable image registration, adaptive radiotherapy, thoracic, computed tomography

## Abstract

**Introduction:**

Deformable image registration (DIR) is a required tool in any adaptive radiotherapy program to help account for anatomical changes that occur during a multifraction treatment. SmartAdapt is a DIR tool from Varian incorporated within the eclipse treatment planning system, that can be used for contour propagation and transfer of PET, MRI, or computed tomography (CT) data. The purpose of this work is to evaluate the registration and contour propagation accuracy of SmartAdapt for thoracic CT studies using the guidelines from AAPM TG 132.

**Methods:**

To evaluate the registration accuracy of SmartAdapt the mean target registration error (TRE) was measured for ten landmarked 4DCT images from the https://www.dir‐labs.com/ which included 300 landmarks matching the inspiration and expiration phase images. To further characterize the registration accuracy, the magnitude of deformation for each 4DCT was measured and compared against the mean TRE for each study.

Contour propagation accuracy was evaluated using 22 randomly selected lung cancer cases from our center where there was either a replan, or the patient was treated for a new lesion within the lung. Contours evaluated included the right and left lung, esophagus, spinal canal, heart and the GTV and the results were quantified using the DICE similarity coefficient.

**Results:**

The mean TRE from all ten cases was 1.89 mm, the maximum mean TRE per case was 3.8 mm from case #8, which also had the most landmark pairs with displacements >2 cm. For contour propagation accuracy, the DICE coefficient results for left lung, right lung, heart, esophagus, and spinal canal were 0.93, 0.94, 0.90, 0.61, and 0.82 respectively.

**Conclusion:**

The results from our study demonstrate that for thoracic images SmartAdapt in most cases will be accurate to below 2 mm in registration error unless there is deformation greater than 2 cm.

## 1 INTRODUCTION

Deformable image registration (DIR) is at the heart of a robust adaptive radiotherapy program. Deformable image registration is needed to manage inter‐ and intrafractional changes of patient anatomy relative to their treatment plans. Examples of these changes include breathing, weight changes, surgeries, disease progression/regression, or simply those caused by variations in imaging setup/position (i.e., arms up vs arms down). Anatomical variation is the major reason why the delivered dose can never be exactly equal to the planned dose. Using a DIR tool in radiation therapy helps achieve a better understanding of the total delivered dose to a patient. For example, trying to adapt a treatment plan to the changes in the target volume as the treatment course progresses, DIR allows two anatomies to be linked together deformably allowing the contours and dose to be transferred from one computed tomography (CT) study to another.

The goal of any DIR algorithm is to produce a deformation vector field (DVF), mapping voxels from a source image to voxels in a target image. There are many different approaches to produce a DVF and are typically defined by three components namely image similarity metric, regularization, and optimization. Image similarity metrics are used by an algorithm to determine how “correct” the registration is at any step. Regularization is how the algorithm produces a realistic DVF based on desired properties, for example conservation of mass or continuity. Optimization is the algorithms approach to combine regularization and image similarity metric to reach an optimal DVF quickly. The characteristics of all of these components define a DIR algorithm and will define its accuracy and efficacy for different imaging modalities and anatomical sites depending on image contrast and deformation type and magnitude.^1^, ^2^

SmartAdapt (V 13.6) is a DIR tool available to Eclipse^TM^ treatment planning system (Varian Palo Alto, CA). It is understood that SmartAdapt is based on an accelerated demons algorithm,^3^ which uses the gradients in image intensity values to drive the registration.^4^ Driven by image intensity, SmartAdapt can perform DIR on CT, CT‐PET, and MRI and propagate contours between different datasets. However, the dose deformation feature, is not currently supported in the software. Varian’s other DIR software, Velocity™, uses a B‐spline driven deformable image registration and provides the dose deformation tool. SmartAdapt software is usually included in Eclipse TPS by default and is of lower cost compared to Velocity. As such, the prospects of using SmartAdapt are attractive to Eclipse^TM^ users for both contour propagation and possibly dose deformation for clinics with limited resources.

The Task Group 132 of the AAPM^5^ has provided guidelines for understanding DIR tools and recommends commissioning, quality assurance, and quality control methods for the clinical use of image registration processes. As per the guidelines, any DIR tool needs to be commissioned before clinical implementation allowing physicists to better understand the fundamental components of the employed DIR algorithm. TG132 suggests primarily a quantitative evaluation of DIR tools through the use of predetermined landmarks to calculate the target registration error (TRE). That report also recommends an independent evaluation of the quality of registration for identifiable features such as organ contours.

There are a number of studies evaluating SmartAdapt for different sites including head and neck,^3^, ^6^ cervical,^7^ and prostate.^8^ Also, there is a report on the evaluation of SmartAdapt using a thoracic phantom.^9^ Those studies can be classified as feature‐based validations since they are comparing manmade contours to automatically propagated contours from SmartAdapt. It should be noted that the validity of contour propagation does not automatically imply the validity of the algorithm for other purposes. An essential element in DIR validation is the evaluation of the interior of contoured volumes using anatomical landmarks. This is of importance when, for example, PET data are registered/deformed to planning CT images or if dose deformation is required. Currently there is no known study performing landmark or contour evaluation of SmartAdapt for thoracic images. In this study we aim to evaluate SmartAdapt using landmarks and contours for thoracic images using TG132 recommendations. This will establish a baseline to provide evidence to support SmartAdapt for CT‐CT registration, contour propagation, CT to PET‐CT deformation as well as for dose deformation.

Thoracic region was chosen for this study due to its unique type of motion and image contrast. Indeed, lung tissue can exhibit large amounts of deformation caused by inflation and deflation within one respiratory cycle. Also, lung cancer patients may undergo plural effusion changing their anatomy, while lung lesions may also exhibit rapid changes during a course of radiotherapy. Thoracic images also exhibit high contrast between bones, soft tissue, and lung providing an excellent contrast to drive the registration algorithm. With all these unique characteristics, DIR has many applications in thoracic CT studies including accounting for tumor volume or anatomy changes during a course of radiation therapy.

## 2 MATERIALS AND METHODS

Based on the recommendations of the TG132 report, a two‐step evaluation including (a) a landmark deformation analysis and (b) an independent assessment of contour propagation by SmartAdapt are studied.

### 2.A Landmark analysis

**FIG. 1:**
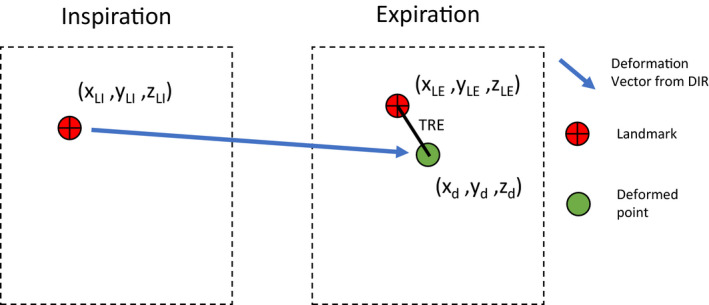
Calculation of target registration error (TRE) for one landmark pair.

Ten 4DCT studies were acquired from the dir‐labs (https://www.dir‐labs.com/)^10^, ^11^ with each study containing 300 landmarks matching the 0% (peak‐inhale) and 50% (peak‐exhale) respiratory phases. These CT datasets are from patients as part of there treatment planning for thoracic malignancies (lung and esophagus) no other selection criteria was considered.^10^, ^11^ This dataset included all of the available 4DCT datasets from dir‐labs and was recommended by TG‐132 for testing the target registration error (TRE) of DIR algorithms. The TRE was calculated for each landmark individually using Eqs. (1) and (2) (Fig. 1).

(1) $$\left(\mathrm{TRE}\right)=\sqrt{{\left(x_{\mathrm{LE}}‐x_{d}\right)}^{2}+{\left(y_{\mathrm{LE}}‐y_{d}\right)}^{2}+{\left(z_{\mathrm{LE}}‐z_{d}\right)}^{2}}$$

(2) $$\left(x_{d},y_{d},z_{d}\right)=\left(x_{\mathrm{LI}},y_{\mathrm{LI}},z_{\mathrm{LI}}\right)+DVF\left(x_{\mathrm{LI}},y_{\mathrm{LI}},z_{\mathrm{LI}}\right)$$

Here, *LI* is the inspiration phase landmark, *LE* is the expiration phase landmark, and *d* is the landmark in the expiration phase produced by the DIR. The mean TRE is calculated by averaging the TRE over all 300 landmarks for each case and is called TRE_mean_. As per AAPM TG132 criteria, the TRE_mean_ should be smaller than the smallest voxel dimension (2 mm)^10^, ^11^ for all ten cases included in this cohort. In addition, TG132 recommends that TRE_mean_ be less than 2mm and the max TRE be less than 5mm, specifically in case number 6.

Mean landmark displacement, *D_LM_*, quantifies the magnitude of deformation for each case and is calculated using Eq. (3). Large *D_LM_* is an indication of large anatomical deformation.

(3) $$D_{\mathrm{LM}}=\sum_{i}^{300}\frac{1}{300}\sqrt{{\left(x_{\mathrm{LE}}^{i}‐x_{\mathrm{Li}}^{i}\right)}^{2}+{\left(y_{\mathrm{LE}}^{i}‐y_{\mathrm{Li}}^{i}\right)}^{2}+{\left(z_{\mathrm{LE}}^{i}‐z_{\mathrm{Li}}^{i}\right)}^{2}}$$

### 2.B Contour propagation accuracy

In this section, we evaluate SmartAdapt’s DIR algorithm by comparing its contour propagation accuracy to human‐made contours in rescanned thoracic patients. We randomly chose 22 thoracic cancer patients from our clinical cohort that had been rescanned in the same anatomical region. Patients were rescanned either during an active course of radiation therapy for adaptation (7 patients) or were rescanned for a new course at or in the vicinity of a previously irradiated site (15 patients). In both scenarios, patient’s anatomy has changed. All patients were treated for thoracic lesions with disease located either within the lung (N = 16) or in the mediastinum (N = 6). For each case, we designated the source as being the older image and the target as being the most recent image set. Each source was deformably registered to its corresponding target using SmartAdapt DIR before contours were propagated from source to target. Those propagated contours (C_P_) were compared to the manual contours (C_M_) using the DICE similarity coefficient. DICE is calculated as the ratio of the volume overlap between C_M_ and C_P_ over their average volume [Eq. (4)]. From Eq. (4), DICE = 1 when there is perfect agreement while DICE = 0 when there is no agreement between two contours.

(4) $$\text{DICE}=\frac{2\left|C_{P}\cap C_{M}\right|}{\left|C_{P}\right|+\left|C_{M}\right|}$$

TG132 guidelines suggest that a DICE larger than 0.8–0.9 indicates a relatively good agreement. Evaluated contours, for the purpose of this study, included the gross target volume (GTV), esophagus, spinal canal, heart, and left and right lungs. The GTV was selected because it is the primary target used in 4DCT scans and encloses a bulk of mass while PTV has a virtual border not defined upon a density gradient. For all 22 pairs of CT datasets the target and esophagus were contoured and peer reviewed by radiation oncologists and the remaining structures were contoured by radiation therapists (lungs, heart, and spinal canal) as per our clinical procedures. Although it is not to intent of this paper to investigate the effects of user variation in contouring we understand some bias may be introduced by not strictly controlling who contoured each image, but we expect this bias to not be significant.

One should note that the relevance of DICE for assessing contours highly depends on the shape and size of the evaluated structure. Structures with larger relative surface area (spinal canal, esophagus) will exhibit larger DICE variation for small changes. However, DICE is less affected for structures with lower relative surface area (lung, heart, prostate) when subject to the same type of change. Based on this, we will modulate our quality criteria and accept a DICE as small as 0.8 for small structures (GTV, spinal canal, and esophagus). For all other structures, we expect the DICE to be larger than 0.9 for large structures (heart and lung) as per TG132. This DICE sensitivity to structure shape and size has been discussed in Deeley et al.^12^

## 3 RESULTS

### 3.A Landmark analysis

**FIG. 2:**
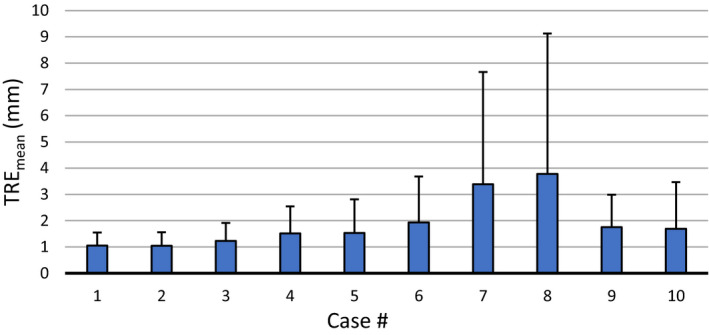
Mean target registration error (TRE_mean_) per study from SmartAdapt. Error bars display one standard deviation.

TRE_mean_ was calculated for each case after a DIR is performed with SmartAdapt and the results are shown in Fig. 2. SmartAdapt achieved a TRE_mean_ < 2 mm (smallest voxel dimension) for all cases except in cases 7 and 8. Averaged over all ten cases, SmartAdapt resulted in a TRE_mean_ of 1.89 mm. For case 6 which is specifically mentioned in TG132, the TRE_mean_ was 1.92mm and TRE_max_ was 10.7 mm.

**FIG. 3:**
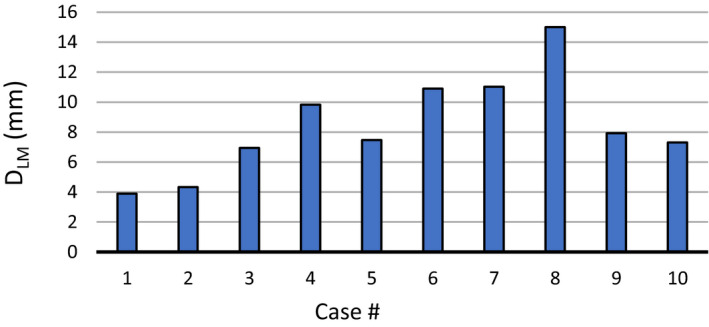
Average landmark displacement (D_LM_) per case.

**FIG. 4:**
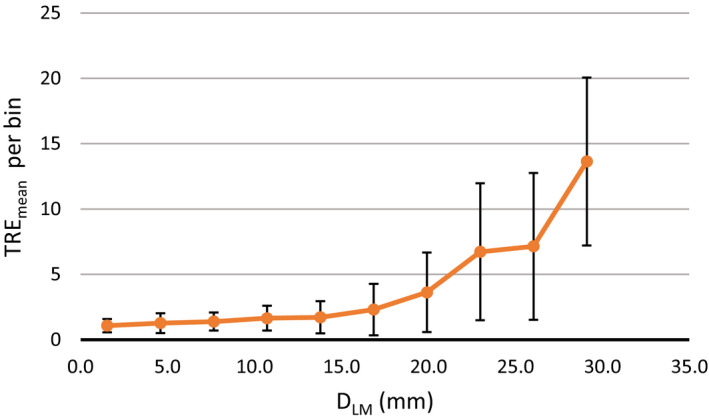
Mean target registration error (TRE_mean_) for landmarks with different amounts of landmark displacement (D_LM_). Each bar represents a different bin, with the landmark displacement representing the bin center. The error bars present one standard deviation.

The D_LM_ is presented in Fig. 3 for all ten cases. It can be seen that cases 4, 6, 7, and 8 show the largest D_LM_, about 10 mm on average. However, the TRE_mean_ is large for cases 7 and 8. To investigate this in‐depth, all 3000 landmarks were extracted from the ten cases and sorted into ten equally spaced bins based on their D_LM_. The TRE_mean_ was then calculated for each bin and displayed in Fig. 4.

**FIG. 5:**
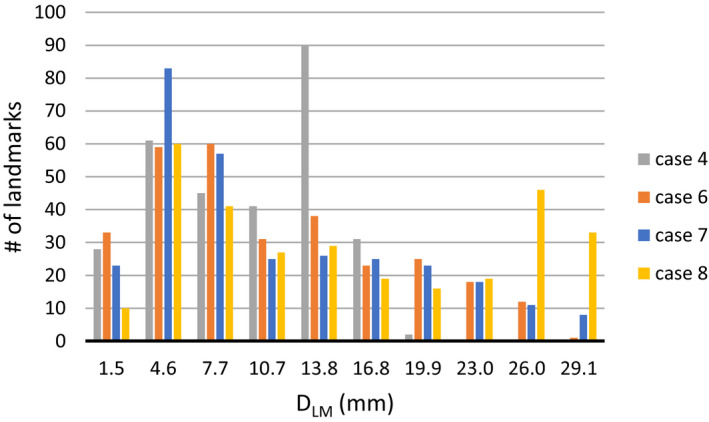
Histogram of landmarks exhibiting different levels of landmark displacement (D_LM_) for cases 4, 6, 7, and 8.

As expected, TRE_mean_ increases with the displacement magnitude but the increase is not linear but rather an exponential behavior can be seen. Figure 5 is breaking down the distribution of landmark displacements per case and cases 4 and 6 have fewer landmarks with displacements above 23 mm. However, cases 7 and 8 have the largest number of landmarks with displacement >23 mm which may partially explain the higher TRE_mean_ in cases 7 and 8.

### 3.B Contour propagation accuracy

The DICE similarity coefficients for GTV, esophagus, spinal canal, heart, and left and right lungs are averaged over all patients and presented in Table 1. For comparison, contour propagation subsequent to a rigid registration is also shown in that table. As expected, a rigid registration produces smaller DICE than DIR for all contours.

**TABLE 1:** Average DICE for eight different structures. N indicates the number of cases used for averaging and one standard deviation is shown in brackets.

	Heart (N = 20)	Left lung (N = 21)	Right lung (N = 21)	GTV (N = 6)	Spinal canal (N = 20)	Esophagus (N = 17)
Rigid only	0.86 (0.07)	0.90 (0.06)	0.87 (0.12)	0.62 (0.22)	0.76 (0.09)	0.54 (0.20)
DIR	0.9 (0.02)	0.93 (0.04)	0.94 (0.02)	0.73 (0.13)	0.82 (0.05)	0.61 (0.12)

Using the modified criteria SmartAdapt performed well for the lungs and heart with an average DICE above 0.90, and spinal canal having an average DICE above 0.80. SmartAdapt DIR did not perform as well for esophagus as shown by an average DICE value of 0.61. This low performance for the esophagus is partially explained by its high relative surface area but additionally, by the embedded uncertainty for contouring the esophagus among different observers.^13^ GTV also showed a low DICE with a mean value of 0.72 which will be discussed in more detail later in the discussion.

## 4 DISCUSSION

**FIG. 6:**
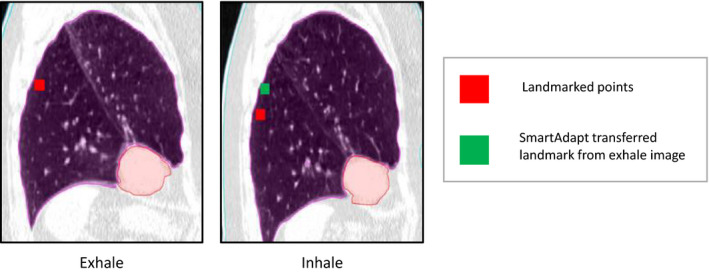
Landmark pair from case 8 with the highest target registration error shown on sagittal view of the exhale and inhale computed tomography studies.

The aim of this work was to determine the clinical viability of the SmartAdapt DIR tool for thoracic CT images. Evaluation was performed using the AAPM TG132, which recommends an average TRE below the maximum voxel dimension. The results for TRE_mean_ criteria passed for all cases except two of them (cases 7 and 8) where TRE_max_ was as large as 26 mm for some of the landmarks. These results demonstrate that for most forms of thoracic deformations, SmartAdapt can be accurate to under 2 mm, which is promising considering the resolution of images in this study was 1 mm with a 2–2.5 mm slice thickness. However, this is not true for points with large displacements, typically greater than 20 mm. It is possible that the TRE could be reduced with higher resolution images, but this was not within the scope of this study. From our results we also demonstrated that it is not simply the average landmark displacement per case that predicts high TRE, but specifically the number of landmarks with large deformations above 20 mm. We hypothesize that the DIR algorithm’s regularization process may heavily penalize deformations >20 mm, which could lead to the large increase in TRE. For example, the landmark with the largest TRE (26.3 mm) from case 8 is shown in Fig. 6 and had an initial D_LM_ of 30 mm in the inferior/posterior direction. This example demonstrates that large internal displacements can result in large TREs, potentially producing a miss registered contour or dose distribution. This emphasizes the importance of TRE analysis for every new DIR algorithm and for every anatomical region where it will be used. To help catch these instances of high TRE, images can be inspected for large deformations above 2 cm especially if they are close to targets or OARs. This analysis should be performed for all anatomical regions where it will be used because a DIR algorithm is dependent on both imaging characteristics and physical deformation which are unique to different regions of the human body.

The dataset used in this study for landmark evaluation came from the https://www.dir‐lab.com/ created from the work from Castillo et al.^10^, ^11^ There are many different articles using the same dataset to test a variety of DIR algorithms including new research DIR algorithms.^14^, ^15^ For this study we compared SmartAdapt only against commercially available algorithms and did not compare it to those research algorithms to keep our conclusions clinically practical. A study by Kadoya et al.^16^ used the same ten cases from https://www.dir‐lab.com/ and evaluated three commercial algorithms namely Raystation, MIM, and Velocity and they compiled their data from 12 different institutions. When the results using SmartAdapt are compared to Kadoya et al.,^16^ SmartAdapt had a lower TRE_mean_ than 11 of 12 institutions which participated in that study. When looking at each case individually, SmartAdapt performed better than the average for every case including case 8. These results help present SmartAdapt as an eligible DIR algorithm for thoracic CT studies on par with other commercial options presented in Kadoya et al.^16^

Feature‐based validation using contours proved SmartAdapt’s DIR to conform to TG‐132 criteria for the heart, lungs, and spinal canal. This argument was not true for the esophagus and the GTV. Our results proved to be similar to other DIR algorithms evaluated in the literature. Peroni et al.^17^ used a B‐spline‐based DIR algorithm registering different phases of 4DCT images and found similar ranges of DICE values for lung 0.89–0.97, heart 0.64–0.93, spinal cord 0.76–0.93, and esophagus 0.42–0.81. Comparing pretreatment to mid‐treatment CT, a study by Hardcastle et al.^18^ obtained mean DICE values for lung at 0.97, cord at 0.9, and esophagus at 0.76 using a demons DIR algorithm. Using the commercial algorithm from MIM (MIM 6.6, MIM software Inc.) a study by Guy et al.^19^ determined ranges of DICE values for lung 0.95–0.97, heart 0.92–0.95, and esophagus 0.65–0.66, when registering between different imaging positions. When compared to the interobserver variability of the manual contouring of these structures presented in McGal et al.^13^ SmartAdapt’s DICE scores were slightly lower on average. The interobserver variability measured by McGal et al.^13^ had DICE scores of 0.86, 0.96, 0.96, 0.91, 0.74 for spinal cord, left and right lung, heart, and esophagus, respectively.

**FIG. 7:**
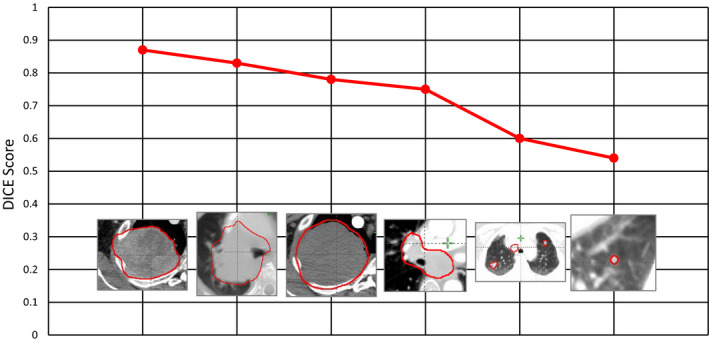
DICE coefficients for the GTV six cases and images of the shape and position of the GTV.

For the GTV, the low DICE scores were somewhat expected given the large interobserver variability on CT. Persson et al.^20^ between radiologists with conformity index values ranging typically from 0.4 to 0.6. The large variation in shape, size, and location of thoracic lesions within the lung can also explain the GTV’s low DICE score and high standard deviation. Also the GTV can be modified by many unpredictable biological processes during the course of a multifraction treatment (including tumor growth or response) which can reduce DIR accuracy. Indeed, the GTV cannot be regarded as a single consistent structure and the average DICE value does not hold a lot of significance because of it. Figure 7 illustrates the GTV shape for each of the six cases involved, with their corresponding DICE scoring. From this figure one can observe that smaller spherical structures within the lung have lower DICE score when compared to larger structures. Thus, contour propagation of small structures that are not clearly visible on CT or exhibit large interobserver variability will likely perform poorly using SmartAdapt’s DIR.

## 5 CONCLUSION

SmartAdapt is a DIR tool available to many Eclipse users, but currently lacks thorough evaluation of its registration accuracy for the clinical use of contour propagation and deformably registering PET or MRI data. The goal of this study was to provide evidence for the future clinical use of SmartAdapt using the TG‐132 recommendations with thoracic CT studies. The landmark analysis measured an average TRE of under 2mm (recommended by TG132) for eight of ten cases, but not for the two cases experiencing large amounts of deformation above 20 mm. Contour propagation using SmartAdapt was within TG‐132 recommendations for lung, heart, and spinal cord contours but not for the esophagus or GTV where manual adjustments may be required. Overall SmartAdapt has shown to be a competent DIR tool for thoracic images within a radiotherapy clinic and should be considered for routine clinical use. Given the favorable results from SmartAdapt in this study, our future work will explore using SmartAdapt for deformable dose transformations within the Eclipse treatment planning system.

## CONFLICT OF INTEREST

The authors have no conflict of interest to report for this study.
